# A mapping review of interventions to address patients who frequently seek care in the emergency department

**DOI:** 10.1186/s12873-024-00970-7

**Published:** 2024-03-27

**Authors:** Ally Memedovich, Benedicta Asante, Maha Khan, Nkiruka Eze, Brian R. Holroyd, Eddy Lang, Sherri Kashuba, Fiona Clement

**Affiliations:** 1 Department of Community Health Sciences, University of Calgary, Calgary, AB Canada; 2 Department of Emergency Medicine, University of Alberta, Edmonton, AB Canada; 3 Department of Emergency Medicine, University of Calgary, Calgary, AB Canada; 4 Emergency Strategic Clinical Network™, Alberta Health Services, Edmonton, AB Canada; 5 O’Brien Institute of Public Health, University of Calgary, Calgary, AB Canada

**Keywords:** Emergency department, Frequent users, Knowledge map, Mapping review

## Abstract

**Background:**

The high utilization of acute care services, particularly emergency departments (ED), continues to be a significant concern for healthcare providers. Numerous approaches have been studied to meet the care needs of patients who frequently seek care in the ED; however, there is no comprehensive review of the current literature base. As such, a current understanding of the interventions initiated within the ED to address the needs of frequent users is required. This mapping review had three objectives: identify the characteristics associated with the need to frequently seek care in the ED; identify interventions implemented to address the needs of this population; and identify gaps in the current evidence base.

**Methods:**

A knowledge map was created by scoping the literature to identify characteristics associated with frequent ED use and interventions implemented to address frequent use. Then, a literature search was conducted to determine what has been implemented by EDs to reduce frequent ED use. The literature was searched from 2013 to January 2023. MeSH terms and keywords were used to identify relevant studies. Studies implementing an intervention for those with characteristics associated with frequent ED use and reporting on ED use were included.

**Results:**

Twenty-three (23) controlled trials and 35 observational studies were included. The most common populations were older adults, those with chronic conditions, and generic “frequent users”. No studies assessed Indigenous Peoples or racial minorities, and few assessed patients with a disability or patients experiencing homelessness. The most common interventions were referrals, care plans, case management, care coordination, and follow-up phone calls. Most studies reported ED revisits, hospitalization, costs, length-of-stay, or outpatient utilization. Few assessed patient or staff perspectives. About one-third of studies (*n* = 24) reported significant reductions in ED revisits.

**Conclusions:**

Similar interventions, mainly focused on care coordination and planning, have been implemented to address frequent use of the ED. There are still significant gaps in the populations that have been studied. Efforts now must be undertaken to study more diverse populations whose care needs are not being met elsewhere and thus frequent the ED often.

**Supplementary Information:**

The online version contains supplementary material available at 10.1186/s12873-024-00970-7.

## Introduction

The high utilization of acute care services, particularly emergency department (ED) visits, continues to be a significant concern for healthcare providers and policymakers internationally. EDs play a crucial role in the healthcare system by providing immediate care to individuals with urgent medical needs 24 h a day, seven days a week. However, a substantial body of research demonstrates that much of the care provided in the ED is for issues that could be addressed outside the ED or in other healthcare settings [1–7].

There is a growing interest in patients who use the ED often. Some patient populations, such as those with chronic pain diagnoses, those with multiple chronic conditions, or older adults, are more likely to utilize EDs frequently [8]. Often people suffering from conditions such as those require highly complex care for health needs stemming from factors such as multimorbidity, psychiatric comorbidities, psychosocial issues, or a combination of these factors [9]. Patients who visit the ED often represent approximately 4.5–8% of ED patients but account for 21–28% of all ED visits [10]. While the definition of “often” varies with some studies suggesting a threshold of 3 or more annual individual patient visits while others employ 12 or more annual visits [11], the need to meet the care needs of patients with different interventions in the ED is commonly noted.

Numerous approaches have been studied over the years to meet the care needs of patients who frequently seek care in the ED. Many interventions have been implemented numerous times since the 1980s [12]. Some interventions are designed to transition patients away from the ED to other settings, such as an electronic medical record (EMR) flag, education, patient navigators, implementing phone lines, expanding primary care hours, and referring patients to relevant service centers that can address their concerns effectively [13–18]. Other interventions have targeted the structure or operation of the ED. These interventions include increased ED staffing, the implementation of care pathways based on risk assessment, the use of screening tools, nurse-led interventions, and integrated care case management within the ED [8, 10, 19]. These interventions also tend to be favoured by ED staff or hospital administrators wanting to reduce high utilization, as it is easier to adopt an intervention in one ED than it is to implement system-wide changes or community-based interventions. So while addressing the needs of those who frequently seek care in the ED will likely require comprehensive, society-wide changes, understanding what ED staff and administrators can do in the meantime is imperative.

### Goals of this investigation

Despite a multitude of research on this topic existing, there is no comprehensive review of the current literature base. As such, a current understanding of the interventions initiated within the ED to reduce ED use in frequent users is required. The overall goal of this mapping review is to identify the characteristics associated with the need to seek care in the ED, identify interventions implemented to reduce unnecessary ED revisits, and to determine if there is any disconnect between who is seeking care and who is being targeted by primary studies.

## Methods

To determine the state of the literature regarding ED-initiated interventions for frequent users, we conducted a mapping review to create an evidence and gap map (EGM). An EGM is a systematic way to identify and display the available evidence and the existing gaps relevant to a specific research question [20, 21]. This review followed the PRISMA reporting guidelines [22, 23].

### Literature search strategy

To get a comprehensive understanding of the state of the literature, a search was conducted. Embase, MEDLINE, CINHAL, Cochrane CENTRAL, and the Cochrane Database of Systematic Reviews were searched for studies published from 2013 to January 18, 2023. This search updated a previously published systematic review conducted in 2013 to capture recently published interventions [24].

The strategies utilized a combination of MeSH terms (e.g., “Emergency Service”, Hospital”, “Patient Readmission”, “Evaluation Study”) and keywords (e.g., “emergency department”, “hotspot”, “intervention study”) to capture interventions of interest. Vocabulary and syntax were adjusted across the databases. The search was limited to English and French language studies. All study designs were retrieved. The search strategy was developed by a research librarian and a peer review of the electronic search strategy was conducted by another research librarian [25]. The full search strategy is available in Appendix A.

Grey literature searches were conducted through the Canadian Agency for Drug and Technologies in Health (CADTH) Grey Matters database, targeted Google searches, and preprint databases including medRixV and Research Square. Canadian provincial health websites were searched for relevant studies or reports. International agency websites including the National Institute for Health and Care Excellence (UK) and Europe PMC were also searched.

Records were downloaded and duplicates were removed using EndNote version 9.3.3 (Clarivate Analytics).

### Study selection

A calibration exercise was conducted by four reviewers on a sample of the retrieved abstracts. After 100% agreement was reached among reviewers, the remaining abstracts were screened in duplicate by two teams of two independent reviewers.

To proceed to full-text review, abstracts had to report on an intervention implemented in the ED with the aim of reducing ED revisits or improving ED care. The columns of the knowledge map created in the previous step were used to determine the characteristics and interventions of interest. The population of interest was adults (18 or older) who were characterized by the authors as “frequent users”, or who had characteristics associated with frequent ED use, such as older adults or those with mental health conditions. Interventions of interest included any intervention that was initiated within the ED. Community-based, hospital-wide, or other interventions were only included if there was an intervention in the ED for these resources. All study designs were included, including qualitative studies that met the other inclusion criteria. Any comparator was considered. The outcome of interest was ED use. Abstracts were excluded if they failed to meet the inclusion criteria, or if they were published in other languages other than English or French. Abstracts selected for inclusion by either reviewer proceeded to full-text review. This initial screen was intentionally broad to ensure that all relevant literature was captured.

A similar calibration exercise was conducted by all reviewers on a sample of the retrieved full-text studies. After 100% agreement was reached among reviewers, full text review was conducted in duplicate by two independent reviewers. Any discrepancies between reviewers were resolved through discussion and consensus. If required, a third reviewer was consulted. Full texts were included if they met the inclusion criteria outlined above. Conference abstracts, case series, reviews, letters, and editorials were excluded.

### Data extraction and analysis

For all included studies, year of publication, country, study design, number of participants, and healthcare practitioner involved in interventions were extracted by a single reviewer using standardized data extraction forms. A second reviewer verified the extracted data. Discrepancies between reviewers during data extraction were resolved through consensus. Additionally, the general population of interest and general intervention utilized were extracted. The data extraction form can be found in Appendix B.

### Determining characteristics associated with frequent use and interventions studied in the literature

A knowledge map was created to determine the common characteristics associated with frequent ED use as well as the interventions commonly used to address these patients. Recent studies, systematic reviews, or scoping reviews on frequent ED use were assessed. The patient characteristics associated with frequent use were extracted. The search continued until saturation of characteristics was achieved. The most common characteristics comprised the columns of the knowledge map. A similar method was undertaken to identify interventions commonly used in the ED to address frequent users. The common interventions comprised the rows of the knowledge map.

The results of the literature review were placed within the knowledge map to identify gaps between the patient characteristics associated with frequent use and the populations studied in the literature. Any intervention or characteristic not identified on the knowledge map but identified in the literature was added.

## Results

### Results of the search

**Fig. 1:**
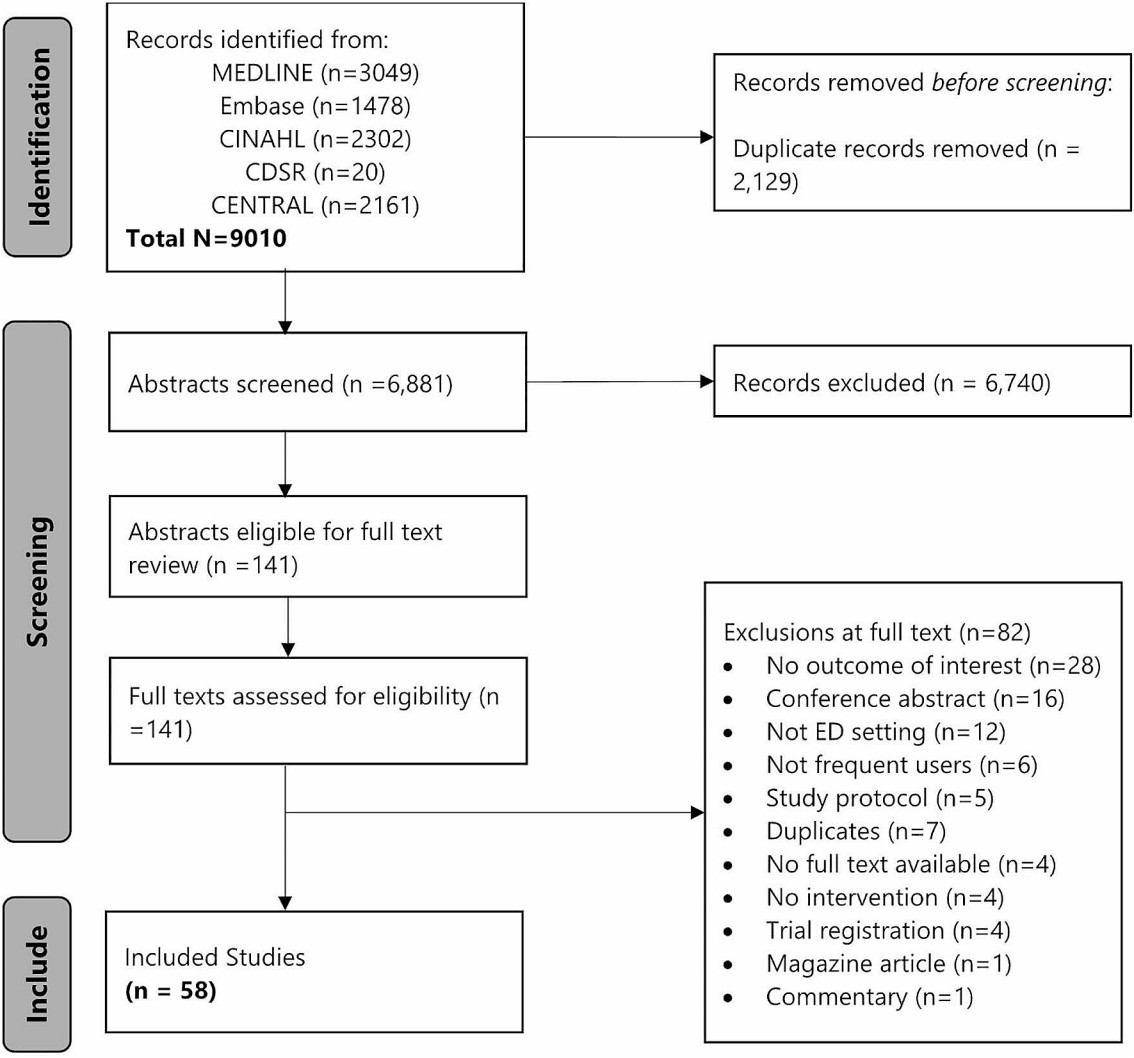
Flowchart of studies

The search strategy yielded 6,881 unique citations, 6,740 of which were excluded after abstract review, Fig. 1. One hundred and forty-one studies proceeded to full-text review. Eighty-two studies were excluded for the following reasons: no outcome of interest (*n* = 26), conference abstract (*n* = 16), not ED setting (*n* = 12), not frequent users (*n* = 6), study protocol (*n* = 5), duplicates (*n* = 5), no full text (*n* = 4), no intervention (*n* = 4), trial registration (*n* = 4), magazine article (*n* = 1), commentary (*n* = 1), Fig. 1.

### Characteristics of included studies

**Fig. 2:**
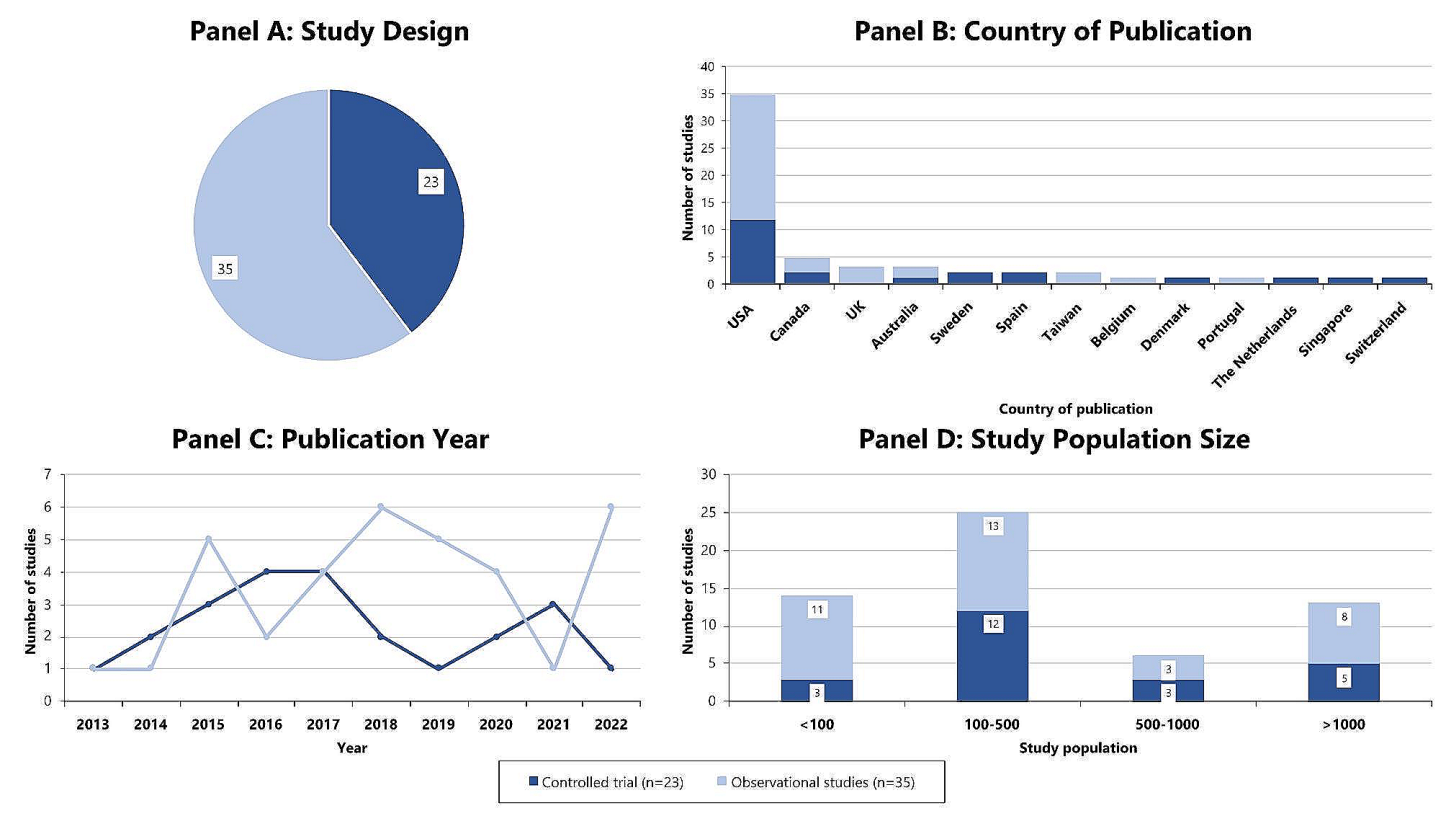
Study characteristics

Overall, 58 full texts were included, including 23 controlled trials, one of which was reported in two publications, and 35 observational studies, Fig. 2. There were no qualitative studies identified. Thirty-five (35) were from the US [14, 16, 26–58], five were from Canada [59–63], three were from each of the UK [64–66] and Australia [67–69], two were from each of Sweden [15, 70], Taiwan [71, 72], and Spain [73, 74], and one was from each of Belgium [75], Denmark [76], Portugal [77], the Netherlands [18], Singapore [78], and Switzerland [79], Fig. 2.

Thirty-six (36) studies used treatment-as-usual as the comparator group, 16 used self-comparator, and four used a control group. Study population size varied widely, ranging from seven participants to over 100,000. Fifteen studies had a study population of less than 100 [14, 26, 30, 34, 36, 41, 44, 47, 51, 57, 62, 65, 66, 68, 71], 24 had a study population of 100–500 [15, 16, 27, 28, 32, 33, 38, 40, 42, 43, 48–50, 53, 55, 58–61, 63, 67, 72, 77, 79], six had a study population of 500–1,000 [31, 45, 64, 74, 78, 80], and 13 had a study population of more than 1,000 [18, 29, 36, 37, 39, 46, 52, 54, 56, 69, 70, 75, 76], Fig. 2.

### Characteristics associated with frequent ED use

Several characteristics associated with frequent ED use were identified by the process to create the knowledge map, Table 1. These were grouped into three main categories: demographic characteristics, patient health and disability status, and social factors. Demographic characteristics included older adults, Indigenous Persons, and racialized groups and ethnic minorities. Patient health and disability status included patients with chronic conditions, patients with mental health conditions, patients with a disability, and patients with a substance use disorder. Social factors included homelessness and social and material deprivation.

**Table 1:** Completed knowledge map

Interventions		Demographic characteristics			Patient health and disability status				Social factors		Unspecified frequent users
		Older adults	Indigenous peoples	Racialized groups and ethnic minorities	Chronic conditions	Mental health conditions	People with a disability	Substance use disorders	People experiencing homelessness	Social and material deprivation	
Continuation of care	Case management	*n* = 2				*n* = 1				*n* = 1	*n* = 6
	Care coordination	*n* = 1			*n* = 1			*n* = 2			*n* = 4
	Care plan	*n* = 2			*n* = 1	*n* = 1		*n* = 1			*n* = 7
	Follow-up phone call	*n* = 4			*n* = 1	*n* = 1					
	Care transition intervention									*n* = 1	
Additional services	Early assessment and intervention	*n* = 1									
	ED physical therapy services	*n* = 2									
	Specialist consult in ED	*n* = 1			*n* = 1						
	ED counselling				*n* = 1						
	Education	*n* = 2			*n* = 2						
	Geriatric assessment	*n* = 4									
	Medication review	*n* = 2									
	Telepsychiatry					*n* = 1					
Warning systems	EMR flag				*n* = 1			*n* = 1			
	Pain contract				*n* = 1						
Services outside the ED	Referral	*n* = 4			*n* = 5		*n* = 1	*n* = 2	*n* = 1	*n* = 1	*n* = 2
	SBIRT							*n* = 1			
Total		*n* = 25	*n* = 0	*n* = 0	*n* = 14	*n* = 4	*n* = 1	*n* = 7	*n* = 1	*n* = 3	*n* = 19

*Some interventions were multi-faceted and therefore are represented multiple times in the knowledge map

In the literature search, the only interventions developed based on patients’ demographic characteristics were interventions for older adults, of which there were 16 studies [18, 27, 29, 35, 36, 40, 45, 54, 56, 67, 69, 72, 74–76, 78]. For patient health and disability status, 12 studies were for those with chronic conditions such as diabetes, chronic pain, or epilepsy [31, 33, 34, 37, 38, 53, 58, 60–62, 66, 73], six for those with substance use disorders [26, 43, 48, 50, 52, 59], three for those with mental health conditions [30, 46, 63], and one for people with a disability [71]. Last, there were four studies developed based on patients’ social factors, three for those with social and material deprivation [32, 44, 51] and one for people experiencing homelessness [57], Table 1.

Additionally, 16 interventions were for “frequent users” as defined by the authors [14–16, 28, 39, 41, 42, 47, 49, 55, 64, 65, 68, 70, 77, 79]. The definition of “frequent user” varied; some authors used patients with a specified number of visits in the last 12 [16, 47, 49, 55, 77, 79] or six months [15, 39, 41, 70], the number of visits during multiple periods of time [14, 28, 42], or the most frequent users over a period [65, 68], such as the top 20 most frequent users in one quarter [68].

The literature search did not identify any interventions developed specifically for Indigenous Peoples, other racialized groups, or ethnic minorities, Table 1.

### Interventions reported in the literature

Many different interventions were identified in the knowledge map, Table 1. These interventions can be broadly grouped into four categories: continuation of care, additional services in the ED, services outside the ED, and warning systems. Continuation of care consists of interventions that assist patients after their encounter in the ED. Additional services in the ED consist of interventions that provide patients with services not typically associated with the ED, such as specialist consultations, physical therapy services, education, or counselling. Services outside the ED included referring patients to services outside the ED, such as outpatient clinics or primary care providers. Last, warning systems consist of systems in place to identify patients as frequent users or limit the services they can receive in the ED, such as EMR flags or limits on opioid prescriptions.

Several interventions were utilized in the literature, and many studies reported multi-faceted interventions, Table 1. Overall, 36 studies reported on continuation of care interventions: 12 reported on care plans [15, 26, 28, 30, 38, 41, 42, 45, 49, 68, 70, 75], nine reported on case management [14, 15, 44, 47, 55, 63, 64, 72, 79], eight reported on care coordination [16, 39, 48, 50, 58, 65, 69, 77], six reported on follow-up phone calls [18, 30, 35, 36, 60, 67], and one reported on a care transition intervention [51]. Seventeen interventions reported on additional services in the ED: four each reported on education [34, 45, 61, 67] and geriatric assessment [72, 75, 76, 78], two each reported on physical therapy services [27, 29], specialist consultations [31, 76], and medication review [27, 74], and one each reported on early assessment and intervention [40], counselling [60], and telepsychiatry [46]. Seventeen reported on services outside the ED, with 16 reporting on referrals [16, 32, 37, 43, 45, 49, 53, 57, 59, 61, 62, 66, 67, 71, 75, 78] and one reporting on Screening, Brief Intervention and Referral to Treatment (SBIRT) [52]. Last, warning systems were reported in three studies, with EMR flags being reported in two studies [43, 53] and pain contracts being reported in one [33], Table 1.

Thirty-six (36) interventions included nurses, 28 included ED physicians, 15 included social workers, 10 included specialists, eight included family doctors or primary care practitioners, and 33 included other healthcare professionals, intervention staff, or volunteers. Other physicians included in interventions were cardiologists, endocrinologists, psychiatrists, geriatricians, and pulmonologists. Specialists included in interventions include physical therapists, pharmacists, and psychologists. Other staff involved include case managers, care coordinators, clinical directors, peer specialists, legal staff, representatives from local insurance organizations, and health navigators.

### Outcomes reported in the literature

**Fig. 3:**
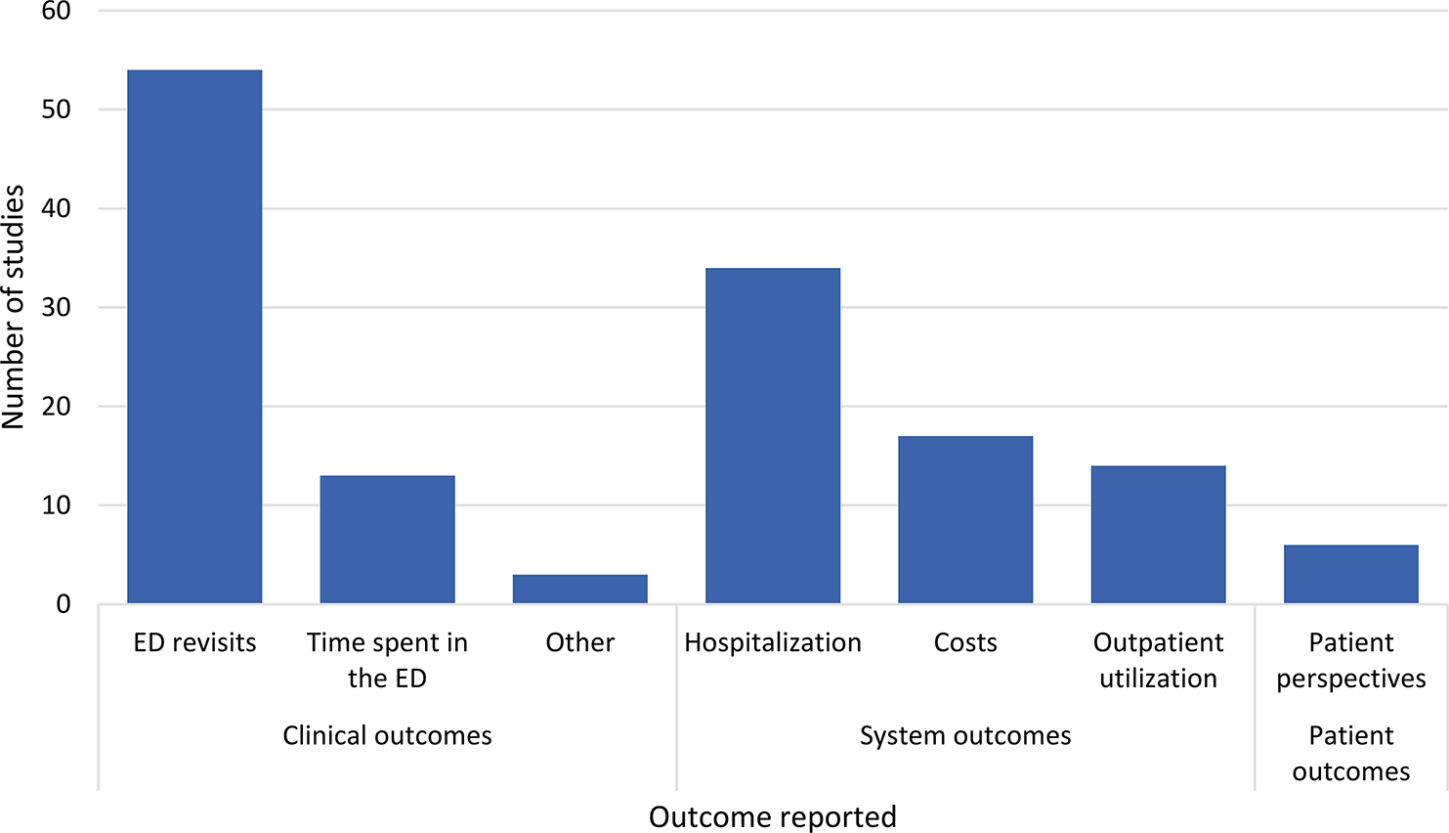
Outcomes reported in the literature

Outcomes reported in this literature can be broadly categorized as system outcomes, clinical outcomes, and patient-reported outcomes, Fig. 3. For clinical outcomes, 54 studies reported ED revisits, 13 reported time spent in the ED, and one each reported the number of diagnostic tests conducted [57], number of referrals provided by ED staff [61], and opioid prescriptions provided by ED staff [50]. Importantly, no studies reported on whether patients’ conditions were improved or worsened. For system outcomes, 34 studies reported on hospitalizations, 17 reported costs, and 14 reported on outpatient utilization. Six studies reported on patient perspectives, though none of these were qualitative. Overall, 24 studies reported significant reductions in ED revisits, 12 reported significant reductions in hospitalization, five reported significant reductions in time spent in the ED, four reported significant increases in outpatient utilization, three reported significant reductions in costs, and one reported significant improvement in patient perspectives.

**Fig. 4:**
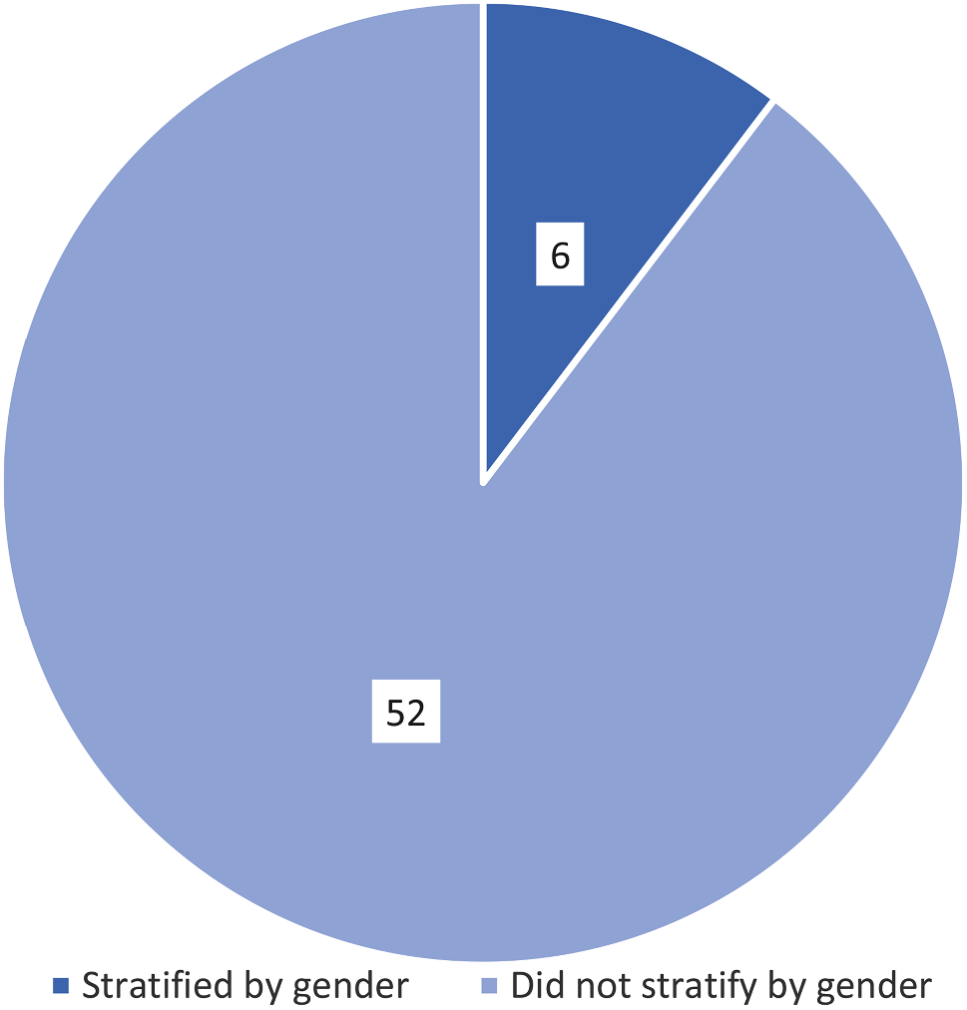
Stratification by gender

Six studies reported results by sex and/or gender [15, 18, 26, 41, 47, 72], Fig. 4. Of these, three were for those who frequently seek care in the ED [15, 41, 47], two were for older adults [18, 72], and one was for those with a substance use disorder [26]. Two were controlled trials [15, 18] and four were observational studies [26, 41, 47, 72]. Two were multi-faceted interventions, assessing case management and care plans [15] and geriatric assessment and case management [72]. The other interventions assessed care plans [26, 41], case management [47], and follow-up telephone calls [18]. Two studies identified significant differences between men and women, with women consistently having higher ED revisit rates and hospital admissions [26, 72]. No other differences were reported. The remaining four studies found no significant differences between men and women. Additionally, no study distinguished between sex and gender and often it was not clear which concept was being assessed.

## Discussion

Overall, 58 studies were identified, including 23 controlled trials and 35 observational studies. Most studies included interventions targeted frequent users as defined by the authors, older adults, and those with chronic conditions. Very few interventions were for marginalized populations, with only a handful for those with mental health conditions, people with a disability, and people experiencing homelessness, and none for Indigenous Peoples and other racialized persons. The types of interventions ranged, with the most popular being referrals, care plans, care coordination, and case management.

There is clearly a disconnect between the populations studied in this literature and the characteristics typically associated with frequent ED use. There were very few interventions for those with mental health conditions and those with social and material deprivation, despite these factors being associated with frequent ED use [24, 81, 82]. In contrast, the most commonly identified characteristics were older adults and those with chronic conditions; while these characteristics are associated with increase ED use, they typically comprise a smaller percentage of ED visits. Further, there were no studies implemented specifically for non-white patients, particularly Indigenous Persons and racialized minorities. It appears, therefore, that those who are at the highest risk of needing to rely on the ED are not typically the focus of ED-initiated interventions. It is likely that interventions for these patients have been implemented outside the ED; however, these patients make up a relatively large portion of ED patients and should be a focus in ED-initiated interventions. Future directions should focus on the gaps identified by this review. Interventions should be developed for the populations missing, particularly Indigenous Peoples, racialized or marginalized patients, patients experiencing homelessness, or patients with a disability or mental illness. Additionally, the interventions that have been trialed should be implemented for these populations and adapted to fit their specific needs. There is a plethora of research demonstrating the need for culturally appropriate and safe care that is tailored to the specific needs of patients, particularly Black and Indigenous patients [83–86]. For example, case management that is developed specifically for Indigenous patients would likely be more successful at meeting patients’ needs than using generic case management strategies. Ultimately, the needs of the patients being cared for should be prioritized.

One major gap in the literature is the types of interventions tried. The most implemented interventions are very similar. For example, care coordination, case management, and care plans all center around ensuring patients’ care is consistent and patients are being supported. Perhaps the literature suggests that these interventions are not as effective as previously suspected, and that while these interventions may have benefits not explored by this literature, other measures are needed to fully address ED frequent use. Further, many of the interventions assessed did not attempt to address the underlying causes of frequent ED use. Some interventions did—many educational interventions focused on self-management and referrals typically referred patients to outpatient centers to learn about their condition—and there were some interventions tailored to very specific populations that attempted to address the underlying needs of patients, such as one intervention for people experiencing homelessness that referred patients to a housing service. Overall, however, most of the interventions were fairly generic and did not attempt to address the barriers faced by patients. Additionally, the characteristics of frequent users can vary based on location [81, 87, 88], so interventions that work in one location may not be appropriate for another. Perhaps more specific, tailored interventions are needed to truly help patients and reduce frequent ED use.

There were some limitations to this review. The first is that community-based or system-wide interventions were excluded. There have likely been interventions developed for the missing populations that were excluded from this review because they were community-based, which would not provide a totally accurate depiction of the interventions trialed to reduce frequent ED use and the populations that have been considered. Additionally, this review only included studies that reported on ED use as an outcome. Though other outcomes were reported, this focus on ED use as an outcome would have limited the number of studies included in the review. There also was not a focus on patient perspectives; qualitative studies would have been included had they reported on ED use as well, but none were identified. Patient perspectives on ED care is important: patients may feel supported by the interventions or may feel they are more in control of their health, which could have other benefits. However, it is important to understand what has been attempted in the ED to understand where further work is needed and understand how EDs can support the patients that do come to the ED.

## Conclusion

There is a wide range of interventions that have been implemented to address several populations associated with frequent use of the ED. Despite the wealth of literature, however, there are still significant gaps in the populations that have been assessed and the interventions that have been utilized across multiple populations. Future work should focus on implementing specific interventions created for marginalized patients, particularly Black and Indigenous patients, and interventions that meet the needs of the patients attending the ED. Additionally, new, innovative interventions should be tried, given the homogeneity of interventions identified in the literature and the lack of standout interventions.

### Electronic supplementary material

Below is the link to the electronic supplementary material.

Supplementary Material 1

Supplementary Material 2

## Data Availability

All data generated or analyzed during this study are included in this published article and its supplementary information files.
